# Integrated 16S rRNA Sequencing and Metabolomics Analysis Reveal the Protective Effects of (E)-Flavokawain A on AOM/DSS-Induced Colorectal Cancer in Mice

**DOI:** 10.3390/nu18020310

**Published:** 2026-01-19

**Authors:** Xin Zhang, Di Wang, Yang Wang, Meimei Wang, Juncheng Wang, Yue Sun, Siman Chen, Xinting Qu, Antong Xia, Hongxin Liu, Jihui Wang, Meng Liu

**Affiliations:** School of Life and Health Technology, Dongguan University of Technology, Dongguan 523808, China; 2023130@dgut.edu.cn (X.Z.); diwang_cpu@163.com (D.W.); wyang@dgut.edu.cn (Y.W.); wmm543@126.com (M.W.); 13680393191@163.com (J.W.); 13650360035@163.com (Y.S.); 15766197536@163.com (S.C.); 15920612132@163.com (X.Q.); 13929355512@163.com (A.X.); liuhongxin@dgut.edu.cn (H.L.); wangjihui@dgut.edu.cn (J.W.)

**Keywords:** (E)-Flavokawain A, colorectal cancer, gut microbiota, metabolism

## Abstract

(E)-Flavokawain A (FKA), the primary chalcone constituent of *Piper methysticum*, exhibits diverse pharmacological properties and holds significant potential for therapeutic development. **Objectives**: This study aims to investigate the anti-colorectal cancer effects and mechanisms of FKA. **Methods**: Using AOM/DSS-induced colorectal cancer models in C57 mice, the research examines the impact of different FKA doses, employing 16S rRNA and metabolomics to explore the potential mechanism. **Results**: The findings indicated that FKA significantly inhibited the progression of colorectal cancer in C57 mice by modulating the composition of the gut microbiota. This modulation involved the suppression of endotoxin secretion by pathogenic bacteria and the concurrent augmentation of beneficial bacteria. Furthermore, in the context of metabolic pathways, FKA regulates lipid metabolism and arachidonic acid metabolism, thereby mitigating the inflammatory transformation associated with colorectal cancer. **Conclusions**: These findings provide valuable insights supporting the potential of FKA as a viable preventive strategy against CRC.

## 1. Introduction

Colorectal cancer (CRC) is a common malignancy with a growing global prevalence [1]. The International Agency for Research on Cancer (IARC) reports that CRC ranks third worldwide in incidence, following breast and lung cancer. In China, the incidence of CRC is also increasing, posing a significant public health challenge for the country. The therapeutic approaches for CRC include surgical resection, chemotherapy, radiotherapy, and more novel interventions such as targeted therapy and immunotherapy. Surgical resection is typically successful in treating early-stage CRC [2]. However, its effectiveness diminishes in cases of advanced or metastatic disease [3]. Chemotherapy and radiotherapy have shown some success in extending survival rates, but their use is often accompanied by significant adverse effects that can greatly affect patient well-being [4]. Additionally, the emergence of drug resistance to chemotherapy poses a considerable obstacle in the clinical management of CRC. Although targeted therapy and immunotherapy have improved treatment outcomes to some extent, their response rates remain limited, and they are associated with immune-related adverse events [5]. Therefore, the advancement of new therapeutic approaches is of significant importance in clinical practice.

The gut microbiota has been identified as a significant factor in the pathogenesis of CRC, with various studies demonstrating a strong correlation between the composition and diversity of the gut microbiota and the incidence of CRC [6]. Specifically, alterations in the gut microbiota, such as changes in the abundance of *Clostridium*, *Fusobacterium*, *Prevotella*, *Fusobacterium nucleatum*, *Enterotoxigenic Bacteroides fragilis*, *Firmicutes*, *Proteobacteria*, *Campylobacter*, *Erysipelotrichia*, and the *Phascolarctobacteriaceae*, have been associated with the development of CRC [7]. The alterations in the gut microbiota play a significant role in the advancement of CRC by inducing inflammation, disturbing the equilibrium of the gut microenvironment, and worsening metabolic irregularities. Therefore, the manipulation of the gut microbiota emerges as a potentially effective therapeutic strategy for mitigating the occurrence of CRC [8]. Interventions for treatment encompass the administration of probiotics and prebiotics to reestablish a healthy gut microbial profile, as well as dietary modifications aimed at enhancing the diversity and functionality of the gut microbiota, ultimately diminishing the likelihood of CRC development.

The widespread availability limited adverse effects, and cost-effectiveness of natural products have led to their extensive use in cancer treatment. The investigation of CRC therapeutics derived from natural sources holds significant clinical importance. *Piper methysticum* [9], a botanical species belonging to the *Piperaceae* family, has been extensively employed in traditional medicinal practices. Recently, it has been incorporated into beverages and dietary supplements aimed at alleviating symptoms of anxiety and stress [10]. Furthermore, a growing body of research indicates that *Piper methysticum* exhibits a diverse array of biological properties, rendering it a potentially valuable dietary adjunct [11]. The investigation of the efficacy of its bioactive constituents holds substantial practical significance. (E)-Flavokawain A (FKA) [12], the primary chalcone found in *Piper methysticum*, has been shown to possess antitumor, anti-inflammatory, antioxidant, and immunomodulatory properties [13,14]. Research has shown that FKA possesses the ability to impede the growth of colon cancer cells, trigger programmed cell death in tumor cells, and hinder tumor-related signaling pathways such as PI3K/Akt and mTOR [15]. Additionally, FKA has been observed to augment the effectiveness of chemotherapeutic medications like 5-fluorouracil and oxaliplatin, potentially by inhibiting multidrug resistance-associated proteins such as P-glycoprotein in tumor cells [16].

Drawing on existing research, this study seeks to employ a comprehensive methodology involving network pharmacology, metabolomics and 16S rRNA technology to thoroughly investigate the impacts and underlying mechanisms of FKA in the management and prevention of CRC. The objective of this endeavor is to establish a solid empirical basis for the future advancement of FKA as a beneficial dietary supplement.

## 2. Materials and Methods

### 2.1. Reagents and Chemicals

(E)-Flavokawain A (CAS: 37951-13-6) was purchased from MedChemExpress (Monmouth Junction, NJ, USA); Azoxymethane (AOM) (98%) was purchased from Aladdin (Shanghai Aladdin Biochemical Technology Co., Ltd., Shanghai, China); Dextran sulfate sodium (DSS) was purchased from MP Biochemicals (Irvine, CA, USA).

### 2.2. AOM/DSS-Induced Colorectal Tumorigenesis and Administration

**Figure 1:**
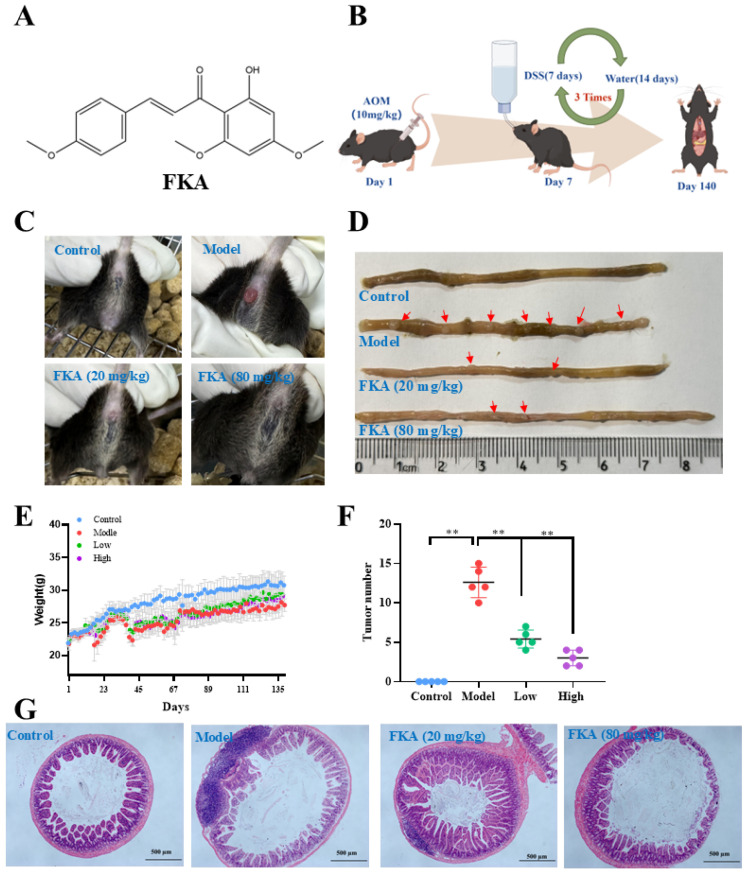
In vivo anti-tumor assessment of FKA in the AOM/DSS model. (**A**) Structural formulas of FKA; (**B**) Schematic illustration of the AOM/DSS mouse model establishment (by FigDraw2.0), ID:AYTAR00385); (**C**) The representative images of external prolapses from the anus in different mice; (**D**) Representative image of the distal colon in different groups; (**E**) Body weight change graph. (**F**) Quantification of the number of tumors in the colon (*n* = 5), ** *p* < 0.01, compared with model group; (**G**) Colon tissue sections of mice stained by H&E (Baton Rouge, LA, USA) in different groups (Scale bar, 500 μm).

The twenty-five C57BL/6 male mice, aged 4 weeks, were procured from Zhuhai BesTest Bio-Tech Co., Ltd. (SCXK(YUE)2020-0051, Zhuhai, China) and housed in the Animal Laboratory of Guangdong Medical University, where they were maintained under controlled conditions of 25 ± 2 °C temperature, 60–70% humidity, and an artificial 12 h light/dark cycle (8 am–8 pm). All experiments were approved by the Ethics Committee of Guangdong Medical University (ethical approval number: GDY2304013, Date: 13 April 2023). The AOM/DSS mouse model was established using a previously reported method. Briefly, mice were administered a single intraperitoneal injection of 10 mg/kg AOM, followed by three cycles of 2.5% DSS in their drinking water for one week, then a two-week recovery period with regular drinking water (Figure 1B). The mice were then randomly assigned to four groups (*n* = 5): the normal group, the AOM/DSS group, the FKA low-dose group (p.o., 20 mg/kg/2d), and the FKA high-dose group (p.o., 80 mg/kg/2d). Furthermore, mice in both the normal and AOM/DSS groups were administered an equivalent volume of water orally. Parameters such as body weight, diarrhea, and hematochezia were closely observed throughout the experimental period. Upon completion of the 19-week period, the mice were euthanized, and the quantity of colorectal tumors present was documented. Various samples including blood, feces, colonic contents, and colorectal tissues were gathered for subsequent analysis.

### 2.3. Histopathological Analysis

The distal 1–2 cm segments of the colorectal tract were excised from each mouse and preserved for histopathological examination using hematoxylin and eosin (H&E) staining techniques. The stained tissue sections were subsequently analyzed visually using a Zeiss Optical Microscope (Oberkochen, Germany) to capture histopathological microscopic imagery. A semi-quantitative histopathological scoring system was employed to evaluate the extent of injury to the colonic epithelium.

### 2.4. 16S rRNA Sequencing of Fecal Bacteria

Fecal samples were collected from three experimental cohorts and stored at −80 °C for future microbiological analysis. Genomic DNA was extracted from the intestinal contents of rodents using the e.z.n.a^®^ Soil DNA Kit (Omega Bio-tek, Norcross, GA, USA). The V3–V4 hypervariable region of the 16S rRNA gene was amplified by PCR using the F338/R806 primer set(Thermo Fisher Scientific, Waltham, MA, USA). The resulting amplicons were processed according to the protocol from Personalbio (Shanghai, China). After quality control to remove low-quality and adapter sequences, the high-quality reads were demultiplexed by index and barcode to assign them to samples. After barcode removal, sequences were processed with QIIME2 for denoising and OTU clustering. ASVs/OTUs were annotated using the Silva database, and microbial composition was visualized at various taxonomic levels. Alpha diversity metrics assessed within-sample diversity, while rarefaction curves evaluated sequencing depth. Beta diversity was analyzed using pairwise distance matrices, NMDS, and PCoA to visualize sample differences. The PERMANOVA test evaluated the statistical significance of species abundance differences. Hierarchical clustering, PCA, and LEfSe were used to identify potential biomarkers. Correlation networks were constructed to analyze species interactions and identify key species through topological indices. Metabolic functions of the microbiota were predicted using 16S rRNA, 18S rRNA, and ITS gene sequencing. Functional profiles were compared to find differentially abundant pathways, and species composition in these pathways was analyzed. Statistical analyses were conducted using Personalbio’s online tools (www.genescloud.cn).

### 2.5. Metabolomics Study on Intestinal Tissue

Analysis was conducted using an UHPLC (1290 Infinity LC, Agilent Technologies, Santa Clara, CA, USA) paired with a quadrupole time-of-flight mass spectrometer (AB Sciex TripleTOF 6600, Framingham, MA, USA). For HILIC separation, a 2.1 mm × 100 mm ACQUITY UPLC BEH Amide 1.7 µm column (Waters, Ireland) was utilized. The mobile phase in both ESI positive and negative modes consisted of A = 25 mM ammonium acetate and 25 mM ammonium hydroxide in water, and B = acetonitrile. The gradient started at 95% B for 0.5 min, decreased linearly to 65% in 6.5 min, then to 40% in 1 min, held for 1 min, and returned to 95% in 0.1 min. The ESI source conditions were: Gas1 and Gas2 at 60, curtain gas at 30, source temperature at 600 °C, and ISVF at ±5500 V. For MS-only acquisition, the *m*/*z* range was 60–1000 Da with a TOF MS scan time of 0.20 s/spectra. For auto MS/MS acquisition, the *m*/*z* range was 25–1000 Da with a product ion scan time of 0.05 s/spectra. The product ion scan was obtained utilizing information-dependent acquisition (IDA) with the high sensitivity mode enabled. The parameters were configured as follows: the collision energy (CE) was set to 35 V with a variation of ±15 eV; the declustering potential (DP) was established at 60 V for positive ions and −60 V for negative ions; isotopes within a 4 Da range were excluded, and up to 10 candidate ions were monitored per cycle.

The raw MS data were converted to MzXML files using ProteoWizard3.0 and then imported into XCMS software. For peak picking, parameters were set to centWave *m*/*z* = 10 ppm, peakwidth = c (10, 60), and prefilter = c (10, 100). Peak grouping used bw = 5, mzwid = 0.025, and minfrac = 0.5. Isotopes and adducts were annotated using CAMERA. Only variables with over 50% nonzero measurements in at least one group were retained in the extracted ion features. Compound identification of metabolites was performed by comparing accurate *m*/*z* values (<10 ppm), and MS/MS spectra with an in-house database established with available authentic standards. Metabolite identification was conducted by comparing *m*/*z* values (within <10 ppm accuracy) and MS/MS spectra to an in-house database of authentic standards.

### 2.6. Statistical Analysis

All data are presented as the mean ± standard deviation from a minimum of three independent experiments. Statistical differences among the groups were evaluated using SPSS software (version 20.0), employing a one-way analysis of variance (ANOVA) for the statistical analysis. Correlations were assessed using the Spearman correlation coefficient. Significant differences were denoted as follows: * for *p* < 0.05, ** for *p* < 0.01, and *** for *p* < 0.001.

## 3. Results

### 3.1. FKA Inhibited CRC in AOM/DSS-Induced Mouse Model

After 19 weeks of continuous administration, the model group mice demonstrated varying degrees of anal prolapse, as illustrated in Figure 1C. Upon dissection, the intestinal tumors were enumerated, revealing a significant increase in tumor count in the model group compared to the normal group. Subsequent administration of FKA resulted in a dose-dependent reduction in tumor count (Figure 1D,F), suggesting that FKA mitigates the proliferation of colon cancer in C57 mice. Histological analysis using hematoxylin and eosin (H&E) staining demonstrated that mice in the model group exhibited a substantial polyp accompanied by inflammatory infiltrate, in contrast to the control group. Treatment with FKA resulted in a dose-dependent reduction in the size of the polyps (Figure 1G).

### 3.2. FKA Modulated the Composition of Gut Microbiota in AOM/DSS-Induced Mice

**Figure 2:**
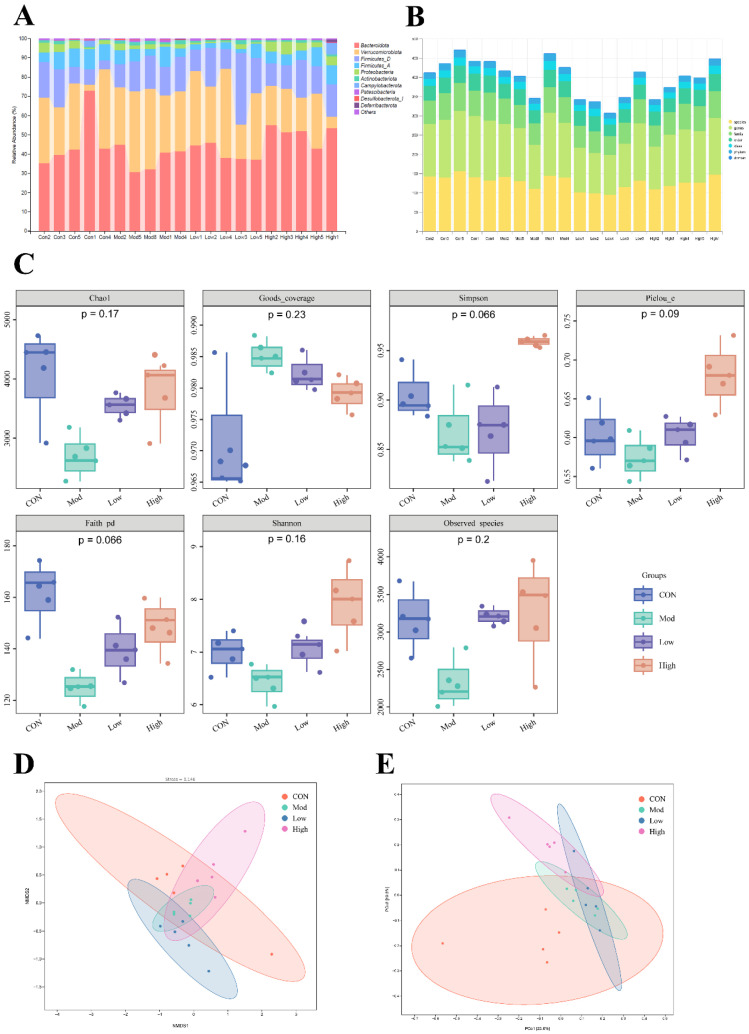
Results of 16S rRNA high-throughput sequencing analysis of gut microbiota. (**A**,**B**) Community composition analysis of gut microbiota; (**C**) Alpha diversity analysis of gut microbiota in different groups; (**D**) NMDS analysis of gut microbiota in mice; (**E**) PCoA analysis of gut microbiota in mice.

The dysbiosis of gut microbiota has been implicated in the pathogenesis and progression of CRC. Consequently, this study employed 16S rRNA sequencing of fecal samples from various groups of mice to examine the compositional differences in microbiota among the Model group, the Normal control group, and the FKA-treated group. The statistical analysis of taxonomic units and the taxonomic composition are presented in Figure 2A,B. The α-diversity indices, encompassing Chao1, Goods coverage, Simpson, Pielou’s evenness (Pielou_e), Faith’s phylogenetic diversity (Faith_pd), Shannon, and Observed species, demonstrated that the richness and diversity of the microbiota in the Model group were significantly reduced in comparison to both the Control group and the FKA-treated group (Figure 2C), suggesting that FKA helps re-establish a more resilient microbial ecosystem, which is typically compromised in CRC. Principal coordinate analysis (PCoA) and non-metric multidimensional scaling (NMDS) demonstrated that FKA treatment induced a dose-dependent alteration in the gut microbiota composition (Figure 2D,E), while this alteration did not align with a restoration towards a baseline or original state in the CON mice. To elucidate the specific microbiota responsible for alterations in microbial composition following administration and to determine whether these changes are associated with the ameliorative effects of FKA on CRC, this study conducted species differentiation and keystone species analysis of the gut microbiota.

**Figure 3:**
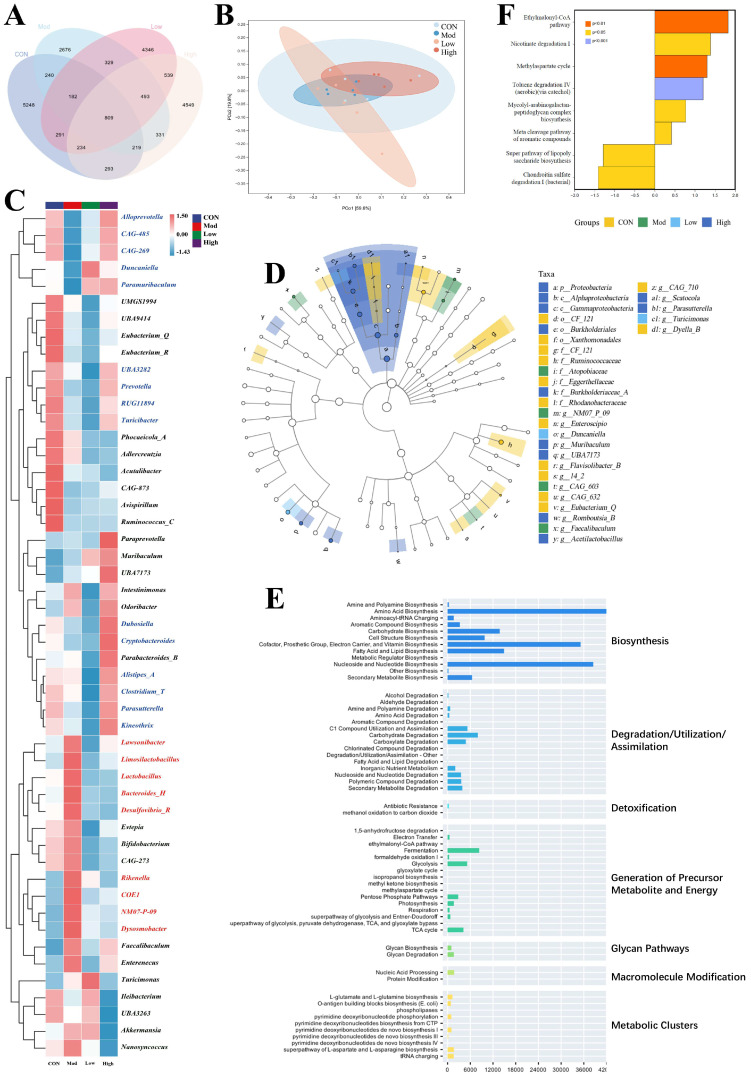
(**A**) Venn diagram of gut microbiota in different groups; (**B**) PCA of gut microbiota; (**C**) Heatmap of gut microbiota in different groups (Red: significantly increased; blue: significantly decreased); (**D**) LEfSe analysis of gut microbiota in different groups; (**E**) KEGG secondary functional metabolic pathway statistics; (**F**) The significantly upregulated metabolic pathways.

As illustrated in the Venn diagram, the FKA-treated group shared 11,571 differentially expressed taxa with the Model group, and 14,143 differentially expressed taxa with the Normal group, while the Model group exhibited 7924 differentially expressed taxa in comparison to the Normal group (Figure 3A). Hierarchical clustering analysis of the top 50 most abundant bacterial genera (Figure 3B,C) demonstrated that the genera *Alloprevotella*, *CAG-485*, *CAG-269*, *Duncaniella*, *Paramuribaculum*, *UBA3282*, *Prevotella*, *RUG11894*, *Turicibacter*, *Dubosiella*, *Cryptobacteroides*, *Alistipes_A*, *Clostridium_T*, *Parasutterella*, and *Kineothrix* exhibited a significant decrease in abundance following DSS/AOM modeling (*p* < 0.01, compared to the control group). Notably, their abundance gradually recovered or even exceeded the levels observed in the control group with increasing concentrations of FKA. In contrast, the genera *Lawsonibacter*, *Limosilactobacillus*, *Lactobacillus*, *Bacteroides_H*, *Desulfovibrio_R*, *Rikenella*, *COE1*, *NM07-P-09*, and *Dysosmobacter* exhibited a significant increase in abundance following DSS/AOM modeling (*p* < 0.01, compared to the Normal group), and their abundance significantly decreased subsequent to FKA administration (*p* < 0.01). Furthermore, certain genera, including *Evtepia*, *Bifidobacterium*, *CAG-273*, *Ileibacterium*, *UBA3263*, *Akkermansia*, and *Nanosyncoccus*, demonstrated expression patterns that were inconsistent with both the Normal and Model groups after FKA administration. The linear discriminant analysis effect size (LEfSe) analysis, which showed that bacteria, including *Scatocola*, *Parasutterella*, *Turicimonas*, and *Dyella B*, are significantly enriched in the high-dose group, potentially correlating with the amelioration of the inflammatory-to-carcinogenic transition in CRC (Figure 3D).

Furthermore, the functional potential of the microbiota constitutes a central aspect of this research. Utilizing known microbial genomic data, we employed PICRUSt2 (Phylogenetic Investigation of Communities by Reconstruction of Unobserved States) analysis on intestinal samples from C57 mice to predict the underlying biomarkers. The statistics of the differentially expressed metabolic pathways among the various treatment groups are depicted in Figure 3E. Notably, the metabolic pathways associated with biosynthesis, particularly those involved in amino acid biosynthesis, fatty acid and lipid biosynthesis, and nucleoside and nucleotide biosynthesis, exhibited the highest expression abundance. The analysis of differential metabolic pathways found that, compared to the model group, the significantly upregulated metabolic pathways after FKA administration included Ethylmalonyl-CoA, Nicotinate degradation I, Methylaspartate cycle, Toluene degradation IV (aerobic) (via catechol), Mycolyl-arabinogalactan peptidoglycan complex biosynthesis pathway, and the Meta cleavage pathway of aromatic compounds (Figure 3F). The significantly downregulated metabolic pathways included the Super pathway of lipopolysaccharide biosynthesis and Chondroitin sulfate degradation I (bacterial). Lipopolysaccharide (LPS) is synthesized by Gram-negative intestinal microbiota. In the model group of mice, the observed upregulation of the lipopolysaccharide biosynthesis superpathway indicates an increased prevalence of these specific bacteria within the intestinal microbiota. These bacteria have the potential to exacerbate the inflammatory-to-carcinogenic transformation of the colon and rectum through mechanisms such as the secretion of pro-inflammatory factors, toxin production, competition for nutrients, and alteration of the intestinal environment. As illustrated in Figure 3C, the abundance of LPS-producing bacteria, including *Lawsonibacter*, *Bacteroides_H*, *Desulfovibrio_R*, and *Rikenella*, was significantly reduced following FKA administration. The administration of FKA markedly diminished the abundance of these bacterial communities and significantly inhibited the super pathway of lipopolysaccharide biosynthesis. This inhibition represents a potential pathway through which FKA modulates the intestinal microbiota, thereby contributing to the amelioration of CRC progression.

### 3.3. FKA Influences Intestinal Metabolomics

**Figure 4:**
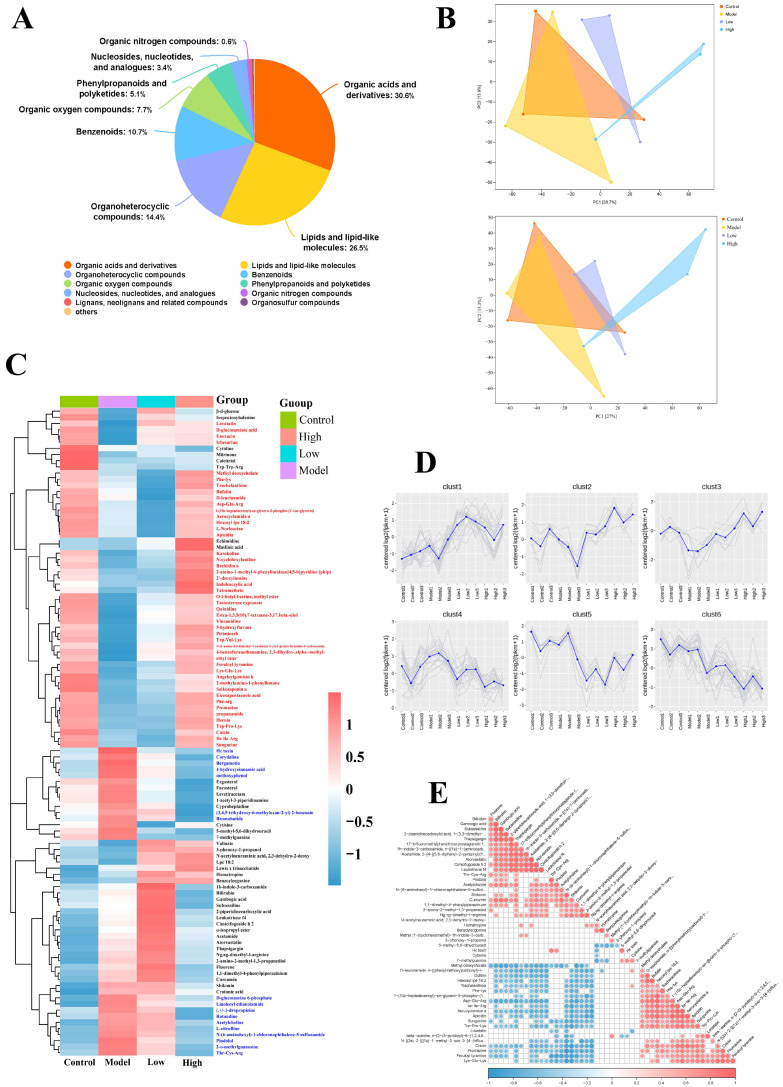
Metabolomic analysis. (**A**) Pie charts showing the proportions of each metabolite class; (**B**) PCA of metabolomic profiles; (**B**) OPLS-DA scattered plots of metabolomic profiles; (**C**) Correlation heatmap of differentially expressed metabolites in different groups (Red: significantly increased; blue: significantly decreased); (**D**) Trend analysis of differentially expressed metabolites in different groups; (**E**) correlation analysis of differentially expressed metabolites in different groups (Red: significantly increased; blue: significantly decreased).

To further investigate the relationship between FKA administration and CRC in mice, as well as its impact on metabolites, this study employed a non-targeted metabolomics analysis on the colon tissues of the mice. Metabolite structures in biological samples were identified by matching retention times, molecular weights (with an error margin < 10 ppm), secondary fragmentation spectra, and collision energies against a comprehensive database. Subsequently, the identified metabolites were categorized and subjected to statistical analysis based on their chemical classification. The relative proportions of each metabolite class were illustrated using pie charts (Figure 4A). The three most abundant classes of metabolites in the mouse intestinal tissue were found to be organic acids and derivatives (28.7%), lipids and lipid-like molecules (26.7%), and organoheterocyclic compounds (16.9%). Furthermore, principal component analysis (PCA) and partial least squares-discriminant analysis (PLS-DA) were employed to derive the principal components for the various experimental groups (Figure 4B). Analysis of the clustering heatmap revealed that, among the top 100 differentially expressed metabolites, 47 metabolites exhibited high expression levels in both the normal and high-dose FKA treatment groups, while being significantly downregulated in the model group. Additionally, 17 metabolites were markedly upregulated following AOM/DSS modeling but showed a gradual downregulation with increasing doses of FKA treatment. These findings suggest that FKA modulates specific metabolic pathways.

### 3.4. Analysis of Metabolic Pathways and Potential Biomarkers of FKA

**Figure 5:**
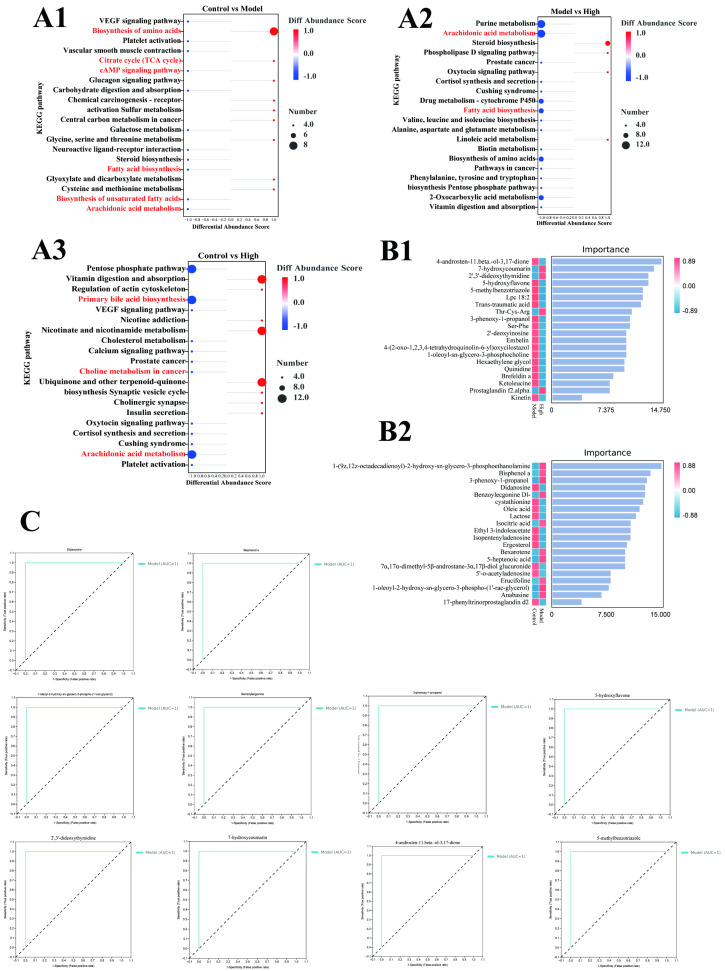
Enrichment analysis of metabolic pathways of potential biomarkers. (**A1**–**A3**) KEGG pathway enrichment analysis of differentially expressed metabolites in different groups, (**A1**): Control vs. Model, (**A2**): Model vs. High, (**A3**): Control vs. High; (**B1**,**B2**) Top 20 metabolites in different groups based on random forests algorithms for biomarker analysis, (**B1**): Model vs. High, (**B2**): Control vs. Model; (**C**) ROC curve of the top 10 potential biomarkers.

To elucidate the specific metabolic pathways regulated by FKA, a correlation analysis of the metabolites was subsequently performed, as illustrated in Figure 4E. KEGG analysis was employed to compare the differential enrichment pathways between distinct groups (Figure 5A1–A3). The analysis of differential abundance revealed that the biosynthesis of amino acids and the tricarboxylic acid (TCA) cycle exhibit a positive correlation in both groups. Conversely, pathways such as the cyclic adenosine monophosphate (cAMP) signaling pathway, fatty acid biosynthesis, the biosynthesis of unsaturated fatty acids, and arachidonic acid metabolism demonstrate a negative correlation. Upon analyzing the metabolite levels, it is evident that post-tumor modeling, the lipid metabolism within the intestinal tissue microenvironment is elevated to sustain tumor growth. This upregulation of lipid metabolism in CRC has been corroborated by clinical evidence. Moreover, the arachidonic acid pathway is significantly upregulated in the model group, suggesting that the AOM/DSS modeling activates the immune response in the organism. In the KEGG enrichment analysis comparing the treatment group and the model group, the expression trends of pathways related to arachidonic acid and lipid metabolism were found to be inversely correlated. This observation suggests that FKA may ameliorate the progression of colorectal inflammation and carcinogenesis in C57 mice by modulating lipid metabolism and inhibiting inflammatory processes.

Utilizing a machine learning model based on the random forest algorithm, we evaluated the significance of intergroup differentially metabolized substances. The top 20 metabolites, ranked by their importance, are illustrated in Figure 5B1,B2. Among the differentially metabolized substances between the normal and model groups, 1-(9Z,12Z-octadecadienoyl)-2-hydroxy-sn-glycero-3-phosphoethanolamine, oleic acid, and 1-oleoyl-2-hydroxy-sn-glycero-3-phospho-(1′-rac-glycerol) are all associated with lipid metabolism (Figure 5B2). This observation suggests that lipid metabolism levels may serve as potential biomarkers for the AOM/DSS model. Isocitric acid, a critical intermediate in the tricarboxylic acid (TCA) cycle, serves as a marker of heightened energy metabolism, a characteristic frequently associated with oncogenesis. The outcomes derived from the machine learning model align with the patterns identified in the KEGG pathway analysis.

In the comparative analysis of differentially metabolized substances between the model group and the high-dose treatment group (Figure 5B1), metabolites such as 4-androsten-11β-ol-3,17-dione, LPC 18:2, and 1-oleoyl-sn-glycero-3-phosphocholine, which are lipid-related, along with peptides and amino acids like Ser-Phe, Thr-Cys-Arg, and ketoleucine, were identified as exhibiting significant changes following FKA administration. These metabolites could potentially serve as biomarkers for distinguishing between the groups. Subsequently, we performed Receiver Operating Characteristic (ROC) analysis on the top five differentially abundant metabolites, prioritized by their importance. As illustrated in the accompanying Figure 5C, the top five metabolites in both the Control vs. Model and High vs. Model comparisons demonstrated a sensitivity of 1 in the ROC analysis. This high sensitivity underscores the significant role these metabolites may play in differentiating between the respective groups.

### 3.5. Correlation Analysis Between Gut Microbiota and Differential Metabolites

**Figure 6:**
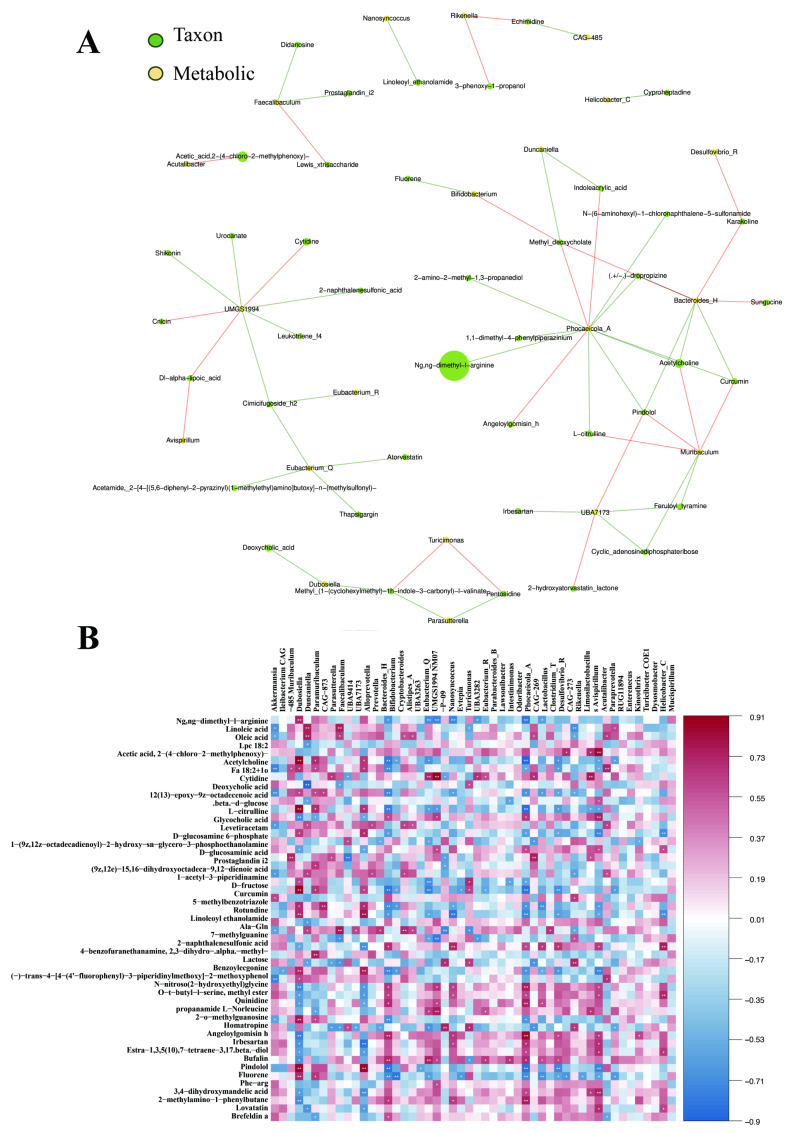
(**A**,**B**) Spearman correlation analysis between differential gut microbiota and key intestinal metabolites. * for *p* < 0.05, and ** for *p* < 0.01.

To further elucidate the potential functional interplay between gut microbiota dysbiosis and intestinal metabolic disturbances, a Spearman correlation analysis was performed between the top 20 significantly altered bacterial genera and the key differential metabolites (Figure 6). The heatmap revealed a distinct and structured correlation pattern, suggesting that FKA-modulated microbiota is closely linked to the restoration of metabolic homeostasis. Specifically, the abundance of beneficial genera, such as *Alloprevotella*, *Prevotella*, and *Muribaculaceae*—which were significantly enriched in the FKA-treated groups—exhibited strong positive correlations with several protective lipid species and organic acids that were upregulated by FKA. Conversely, genera that were overrepresented in the AOM/DSS model group, including *Lawsonibacter*, *Desulfovibrio*, and *Mucispirillum*, showed robust positive associations with pro-inflammatory metabolites, particularly those involved in the arachidonic acid metabolism pathway. Furthermore, these potentially pathogenic taxa exhibited significant negative correlations with the metabolites that were restored by FKA treatment.

## 4. Discussion

CRC is a complex tumor with an unclear pathogenesis, and treatment strategies are evolving to enhance patient outcomes [1]. Research indicates that gut microbiota significantly influences CRC development, with changes in its composition linked to CRC occurrence, potentially impacting the immune response and tumor environment [6]. FKA, a flavonoid compound extracted from natural plants, has demonstrated notable anti-inflammatory properties and significant therapeutic efficacy across various tumor types [14]. However, its potential role in the treatment of CRC remains inadequately understood, necessitating a comprehensive investigation into the mechanistic actions of FKA in CRC therapy.

Our findings indicate a significant reduction in the abundance of *Alloprevotella*, *CAG-485*, *CAG-269*, *Duncaniella*, *Paramuribaculum*, *UBA3282*, *Prevotella*, *RUG11894*, *Turicibacter*, *Dubosiella*, *Cryptobacteroides*, *Alistipes_A*, *Clostridium_T*, *Parasutterella*, and *Kineothrix* in the AOM/DSS-treated group of mice, which was ameliorated following the administration of FKA. The abundance of *Alloprevotella* is significantly reduced in clinical patients with CRC, and its dysregulation may contribute to elevated inflammatory responses, which are directly implicated in the pathogenesis of CRC [17]. Monitoring *Alloprevotella* levels could aid early CRC diagnosis and inspire personalized treatments. Future studies should investigate its specific role in CRC to enhance its use as a clinical biomarker. *CAG-269* has been linked to the development of ulcerative colitis (UC) and is notably upregulated during the remission phases of UC [18], suggesting its potential role in modulating the progression from inflammation to cancer. *Prevotella*, recognized as a probiotic, is believed to ameliorate gastrointestinal complications in CRC patients [19]. Furthermore, as a targeted probiotic, *Prevotella* may enhance the production of short-chain fatty acids by reestablishing gut microbiota equilibrium, thereby contributing to the overall health improvement of patients [20]. In AOM/DSS mice, the bacteria *Lawsonibacter*, *Limosilactobacillus*, *Lactobacillus*, *Bacteroides_H*, *Desulfovibrio_R*, *Rikenella*, *COE1*, *NM07-P-09*, and *Dysosmobacter* were significantly increased but decreased after FKA treatment. *Bacteroides_H* are capable of secreting bile salt hydrolase, which is considered to potentiate the development of CRC [21]. *Lawsonibacter* is linked to irritable bowel syndrome [22], and *Desulfovibrio_R* is a sulfate-reducing bacterium that mainly produces hydrogen sulfide (H_2_S) [23]. H_2_S exhibits toxicity towards the intestinal epithelium and has the potential to compromise intestinal barrier integrity by inducing inflammatory and oxidative stress responses within intestinal epithelial cells, thereby resulting in heightened intestinal permeability. Furthermore, H_2_S may contribute to host metabolic dysregulation by inhibiting the secretion of the gut hormone glucagon-like peptide-1 (GLP-1) [24].

In metabolic analysis, both the control and model groups show a positive correlation in amino acid biosynthesis and the TCA cycle. However, the cAMP signaling pathway, fatty acid biosynthesis, unsaturated fatty acid biosynthesis, and arachidonic acid metabolism exhibit a negative correlation. Biosynthetic processes play a crucial role in CRC development. Since amino acids are essential for cell growth, disruptions in their biosynthesis can cause protein synthesis imbalances, potentially leading to uncontrolled tumor growth [25]. This phenomenon is particularly evident in cancer cells, which often exhibit altered metabolic pathways to support their enhanced proliferation. For instance, the Myc oncogene has been shown to regulate the synthesis of proteins and nucleic acids by controlling a critical rate-limiting enzyme, phosphoribosyl-pyrophosphate synthetase 2 (PRPS2) [26]. This enzyme not only enhances nucleotide biosynthesis but also integrates it with protein synthesis, highlighting a critical intersection exploited by cancer cells for their survival and proliferation [27]. Specific amino acids, such as proline, are vital for the tumor growth of certain cancers. The deprivation of proline can lead to unresolved endoplasmic reticulum stress and interfere with mTORC1-dependent tumor growth, underscoring its significance in maintaining cellular homeostasis in cancer cells [28]. Amino acid biosynthesis plays a crucial role in cancer progression, influenced by amino acid availability and metabolic adaptation. The tumor microenvironment (TME) disrupts amino acid metabolism, particularly tryptophan and arginine, leading to immune suppression and tumor growth [29]. Elevated catabolic enzymes in the TME create an immunosuppressive setting, supporting tumor expansion and therapy resistance. Thus, altered amino acid biosynthesis hampers protein synthesis and fosters metabolic and immune changes that enable tumor proliferation. Addressing these metabolic vulnerabilities may provide novel therapeutic avenues for cancer treatment.

Fatty acids and lipids constitute essential elements of cell membranes, and disruptions in lipid metabolism can compromise membrane integrity and signaling pathways, thereby potentially facilitating cancer cell proliferation and metastasis [30]. Consequently, perturbations in these metabolic pathways may facilitate the onset and progression of CRC by supplying essential substrates and energy required for tumor proliferation, as well as by modulating cellular mechanisms that govern cell survival and apoptosis [31,32]. In this study, the lipid metabolism levels in mice subjected to the AOM/DSS treatment exhibited a negative correlation when compared to both the normal group and the group receiving FKA administration. Analysis of differential metabolites revealed that the significantly reduced metabolites following FKA administration were predominantly lipid-related, such as 4-androsten-11β-ol-3,17-dione, LPC 18:2, and 1-oleoyl-sn-glycero-3-phosphocholine.

Arachidonic acid metabolism plays a crucial role in tumor development and inflammation, particularly in CRC [33]. The metabolism of arachidonic acid leads to the production of various bioactive lipids, including eicosanoids, which are known to influence inflammatory responses and cancer progression [34]. For instance, studies have shown that the arachidonic acid-derived eicosanoids, such as prostaglandins and leukotrienes, can promote tumorigenesis by enhancing cell proliferation and survival while also modulating the immune response in the tumor microenvironment [35]. In CRC, the dysregulation of arachidonic acid metabolism has been linked to increased levels of pro-inflammatory eicosanoids, which can contribute to chronic inflammation—a recognized risk factor for cancer development [36]. For example, the upregulation of the prostaglandin E2 (PGE2) biosynthetic pathway has been observed in CRC patients, correlating with tumor progression and macrophage infiltration [37]. Furthermore, the inhibition of arachidonic acid metabolism has been shown to decrease tumor cell invasion and matrix metalloproteinase expression, suggesting that targeting this metabolic pathway could be a potential therapeutic strategy in managing CRC [38]. FKA impedes arachidonic acid metabolism, thereby modulating the interaction between inflammation and tumorigenesis in CRC. This mechanism may significantly contribute to its capacity to attenuate the inflammatory response induced by toxins such as LPS and H_2_S secreted by the gut microbiota, ultimately mitigating the progression from inflammation to cancer.

Despite the insights provided by our multi-omics approach, several limitations remain. First, while 16S rRNA and metabolomics revealed strong correlations, the direct causal relationship between specific microbial shifts and tumor inhibition was not established through fecal microbiota transplantation (FMT). Second, the functional roles of the identified metabolic pathways (e.g., arachidonic acid metabolism) require further validation at the protein level using Western blot or qPCR for key enzymes like COX-2. Lastly, the sample size (n = 5) and the lack of in vitro human cell line validation mean that the identified biomarkers should be considered preliminary. Future research will focus on germ-free animal models and proteomic analysis to further elucidate FKA’s molecular targets. Further research should incorporate targeted proteomic or phosphoproteomic analyses to elucidate the involvement of these pathways in FKA-mediated anti-cancer effects. Moreover, while our study highlighted the role of FKA in reducing lipopolysaccharide (LPS)-producing bacteria and suppressing inflammatory pathways, the direct causal relationship between specific microbial shifts and tumor inhibition remains to be fully established. Future experiments involving fecal microbiota transplantation or germ-free animal models could help validate the functional contribution of gut microbiota to the therapeutic efficacy of FKA. Finally, the translational potential of our findings to human clinical settings requires further validation through in vitro human cell line studies and prospective clinical trials to assess the safety, bioavailability, and efficacy of FKA in CRC.

## 5. Conclusions

The study showed that FKA effectively improved intestinal flora imbalance in AOM/DSS-modeled mice by reducing harmful bacteria and increasing probiotics, thereby strengthening the intestinal barrier. Metabolomic analysis suggested that FKA may influence lipid and arachidonic acid metabolism pathways in the intestinal tissue. In short, FKA serves as a promising lead compound for the prevention of CRC through the modulation of the gut-metabolism axis.

## Data Availability

The data presented in this study are available on request from the corresponding author due to privacy.
